# Integrative analysis of DNA methylation and gene expression reveals hepatocellular carcinoma-specific diagnostic biomarkers

**DOI:** 10.1186/s13073-018-0548-z

**Published:** 2018-05-30

**Authors:** Jinming Cheng, Dongkai Wei, Yuan Ji, Lingli Chen, Liguang Yang, Guang Li, Leilei Wu, Ting Hou, Lu Xie, Guohui Ding, Hong Li, Yixue Li

**Affiliations:** 1 Department of Bioinformatics and Biostatistics, School of Life Sciences and Biotechnology, Shanghai Jiao Tong University, Shanghai, China; 2 Key Lab of Computational Biology, CAS-MPG Partner Institute for Computational Biology, Shanghai Institutes for Biological Sciences, Chinese Academy of Sciences, Shanghai, China; 3 Basepair biotechnology Co. LTD, Suzhou, China; 4 Department of Pathology, Zhongshan Hospital, Fudan University, Shanghai, China; 5 Shanghai Center for Bioinformation Technology, Shanghai Academy of Science and Technology, Shanghai, China

**Keywords:** Hepatocellular carcinoma, Methylation, CpG island methylator phenotype, Gene regulation, Specific diagnostic biomarker

## Abstract

**Background:**

Hepatocellular carcinoma (HCC) is the one of the most common cancers and lethal diseases in the world. DNA methylation alteration is frequently observed in HCC and may play important roles in carcinogenesis and diagnosis.

**Methods:**

Using the TCGA HCC dataset, we classified HCC patients into different methylation subtypes, identified differentially methylated and expressed genes, and analyzed *cis*- and *trans*-regulation of DNA methylation and gene expression. To find potential diagnostic biomarkers for HCC, we screened HCC-specific CpGs by comparing the methylation profiles of 375 samples from HCC patients, 50 normal liver samples, 184 normal blood samples, and 3780 samples from patients with other cancers. A logistic regression model was constructed to distinguish HCC patients from normal controls. Model performance was evaluated using three independent datasets (including 327 HCC samples and 122 normal samples) and ten newly collected biopsies.

**Results:**

We identified a group of patients with a CpG island methylator phenotype (CIMP) and found that the overall survival of CIMP patients was poorer than that of non-CIMP patients. Our analyses showed that the *cis*-regulation of DNA methylation and gene expression was dominated by the negative correlation, while the *trans*-regulation was more complex. More importantly, we identified six HCC-specific hypermethylated sites as potential diagnostic biomarkers. The combination of six sites achieved ~ 92% sensitivity in predicting HCC, ~ 98% specificity in excluding normal livers, and ~ 98% specificity in excluding other cancers. Compared with previously published methylation markers, our markers are the only ones that can distinguish HCC from other cancers.

**Conclusions:**

Overall, our study systematically describes the DNA methylation characteristics of HCC and provides promising biomarkers for the diagnosis of HCC.

**Electronic supplementary material:**

The online version of this article (10.1186/s13073-018-0548-z) contains supplementary material, which is available to authorized users.

## Background

Hepatocellular carcinoma (HCC) is the sixth most common cancer and the third leading cause of cancer deaths in the world [1]. Most cases of HCC occur in developing countries, such as China, and the leading cause of HCC is chronic infection with hepatitis B virus (HBV); in contrast, the main cause in developed countries, such as the USA, is infection with hepatitis C virus (HCV) [2]. Other risk factors for developing HCC include exposure to aflatoxin, excessive alcohol consumption, tobacco smoking, and diabetes [1]. After being affected by one or more of these risk factors, both genetic and epigenetic alterations will emerge, which may result in the activation of oncogenes and the inactivation of tumor suppressor genes, leading to the occurrence of hepatocellular carcinoma. The 5-year survival rate is > 70% if patients are diagnosed at an early stage [3], while the 5-year survival rate decreases to approximately 10% for advanced HCC patients [4]. Therefore, early detection of HCC is important for increasing the chances for effective treatment and improving the survival rate.

Alpha-fetoprotein (AFP) combined with ultrasonography is a widely used method for the screening and diagnosis of HCC. Marrero et al. [5] reported the diagnostic performance of serum AFP when using a cut-off of 20 ng/mL. Its sensitivity is 59% and specificity 90% for all HCC patients. Additionally, the sensitivity is 53% and the specificity 90% for early-stage HCC [5]. Due to the lack of diagnostic accuracy, the American Association for the Study of Liver Diseases and the European Association for the Study of the Liver do not recommend AFP for HCC diagnosis [6, 7]. The development of omics technologies has allowed researchers to choose a single molecule or a panel of multiple molecules as potential diagnostic biomarkers. Des-γ-carboxy prothrombin (DCP) is a promising serum biomarker. It achieved 74% sensitivity and 70% specificity in all HCC patients, as well as 61% sensitivity and 70% specificity for early-stage HCC at the level of 150 mAU/mL [5]. Another serum biomarker, Dickkopf-1 (DKK1), has similar sensitivity (~ 70%) and specificity (~ 90%) in all HCC patients and for early-stage HCC at a cut-off of 2.153 ng/mL [8]. Although many candidate biomarkers have been reported, few of them are currently used in clinical practice. More effective biomarkers are urgently needed to increase the accuracy of HCC diagnosis.

DNA methylation alteration has been observed in various cancers and is considered to be a cause of carcinogenesis. Global hypomethylation is frequently seen in highly and moderately repeated DNA sequences and plays a key role in chromosomal instability [9, 10]. Hypermethylation in gene promoter regions, such as in tumor suppressor genes, is usually related to gene silencing [9, 11]. Some DNA methylation is involved in the early stage of carcinogenesis, such as RASSF1A in ovarian cancer [12]. Additionally, DNA methylation is relatively stable over time [13] and can be non-invasively detected in blood. Therefore, DNA methylation has a great potential to become an early diagnostic biomarker of cancers. An increasing number of methylation-based biomarkers have been developed to aid in the early diagnosis of cancers [14]. The FDA-approved “Epi proColon test” is based on the SEPT9 promoter methylation status in the plasma. This diagnostic test had a sensitivity of 36.6 to 95.6% and a specificity of 81.5 to 99.0% for colorectal cancer [15]. Zheng et al. [16] reported that using the DNA methylation of ten CpGs could achieve good performance to discriminate tumors from normal tissues in HCC patients, with a sensitivity of more than 86% and a specificity of almost 100%. Xu et al. [17] found that the circulating tumor DNA (ctDNA) methylation of another ten CpGs could also discriminate HCC patients from healthy individuals with a sensitivity of more than 83% and a specificity of more than 90%. Both CpG sets could be good biomarkers for the diagnosis of HCC, but neither of these research groups considered whether other cancer types could have similar methylation alterations; hence, these biomarkers may not be HCC-specific, and specific biomarkers are absent and needed.

In this study, we first classified HCC patients into different methylation subtypes and analyzed the *cis*- and *trans*-regulation of DNA methylation and gene expression. Then, we identified six HCC-specific methylation biomarkers by comparing HCC with normal livers and other cancer types. The combinations of two and six markers achieved 84.8–92.0 and 90.9–92.4% sensitivity and 97.0–100% and 97.0–100% specificity, respectively, in three independent datasets.

## Methods

### Data preparation

DNA methylation, gene expression, and clinical HCC data were collected from The Cancer Genome Atlas (TCGA) project (https://portal.gdc.cancer.gov/). The methylation level of CpGs was represented as β values (375 HCC and 50 normal; β = Intensity of the methylated allele (M)/[Intensity of the unmethylated allele (U) + Intensity of the methylated allele (M) + 100], ranging from 0 to 1) [18]. Gene expression was defined using the raw read count or log2 transformed normalized count (369 HCC and 41 normal). Moreover, the methylation levels for another ten tumor types were collected from TCGA: BLCA (409 tumor, 21 normal), BRCA (774 tumor, 82 normal), COAD (292 tumor, 38 normal), GBM (126 tumor, 2 normal), HNSC (523 tumor, 45 normal), KIRC (316 tumor, 160 normal), LUAD (455 tumor, 32 normal), LUSC (365 tumor, 41 normal), READ (95 tumor, 7 normal) and UCEC (425 tumor, 46 normal), which have both tumor and normal tissues.

Additionally, four methylation array datasets were collected from the Gene Expression Omnibus (GEO) database: GSE69270 [19] (blood of 184 young Finns), GSE54503 [20] (66 paired HCC and adjacent normal), GSE89852 [21] (37 paired HCC and adjacent normal), and GSE56588 [22] (224 HCC, nine cirrhotic, and ten normal). The array platform was the HumanMethylation450 BeadChip (GPL13534). The CpG annotations were downloaded from GEO.

### CpG island methylator phenotype

To find CIMP in HCC, we selected CpGs in the promoter region that have a high standard deviation (SD > 0.2) of the methylation level in 375 tumor tissues and a low methylation level (mean β value < 0.05) in 50 normal tissues, similar to the results of previous studies [23, 24]. K-means-based consensus clustering was performed using the R package ConsensusClusterPlus [25]. Overall survival of the CIMP group and other groups was estimated using the Kaplan-Meier method. Fisher’s exact test was performed to associate the clinical characteristics with each cluster.

### Differential analysis of DNA methylation and gene expression

Fifty of the 375 patients from TCGA have both HCC and normal methylation profiles, and the paired HCC and normal methylation data were used for differential methylation analysis. CpGs with more than 10% missing values were removed. The remaining missing values were imputed with the Bioconductor package impute. Then, a paired *t*-test was used to identify differentially methylated CpGs between the tumor and adjacent normal tissue. *P* values were adjusted using the false discovery rate (FDR) method. CpGs in chromosomes X and Y were ignored. The CpGs with an FDR less than 0.05 and an absolute value of the β difference greater than 0.2 were considered to be differentially methylated. When a CpG mapped to more than one gene, the first gene was taken as the reference.

Of the 50 patients from TCGA, 41 have both HCC and normal expression profiles, and the paired HCC and normal expression data were used for differential expression analysis. The Bioconductor package edgeR [26] was used to identify differentially expressed (DE) genes from raw read counts. Genes with an FDR less than 0.05 and an absolute value of log_2_ (fold change) greater than 1 were considered to be differentially expressed.

### Correlation between DNA methylation and gene expression

The 369 tumor samples with matched methylation and expression data were used for correlation analysis. First, we investigated the correlation between DNA methylation and gene expression (*cis*-regulation). As one gene contains multiple CpGs, Pearson correlation coefficients were calculated between the expression value and the methylation level of each CpG site. Correlation was significant if the correlation coefficient was greater than 0.3 and FDR was less than 0.05. Second, we investigated the correlation of one gene’s methylation and another gene’s expression (*trans*-regulation) using a similar method. Only differentially expressed genes were used to analyze *trans*-regulation, and the DNA methylation was focused on CpGs that were located simultaneously in differentially methylated and differentially expressed genes.

### Identification of candidate diagnostic biomarkers

TCGA datasets were used to screen potential methylation sites as diagnostic biomarkers of HCC. First, 50 paired HCC and normal samples were compared to select hypermethylated CpGs of low-expression genes in HCC. Second, 375 HCC and 50 normal tissues were compared. CpGs without significantly different methylation were filtered out. Third, 375 HCC tissues were compared with blood samples from individuals without HCC (GSE69270); we removed CpGs that had higher average methylation levels in blood than in HCC tissues. Fourth, HCC-specific hypermethylated sites were selected by removing CpGs whose mean methylation levels were higher than 0.1 in tumor or normal samples of another ten tumor types. The remaining CpGs were candidate diagnostic biomarkers of HCC. Finally, information gain-based feature selection was used to decrease the number of candidate diagnostic biomarkers.

### Evaluation of candidate diagnostic biomarkers

The TCGA HCC dataset was taken as the training set, while three other independent datasets (GSE54503, GSE89852, and GSE56588) were used as test sets. A logistic regression model was built based on the methylation levels of the candidate diagnostic biomarkers. This model was used to predict the tumor and normal samples. Sensitivity and specificity were calculated to evaluate the accuracy of the prediction model. Modeling and prediction were performed in the data mining tool WEKA [27].

### Bisulfite sequencing PCR experiments

Surgical biopsies were collected from ten Chinese patients diagnosed with HCC. This study was approved by the ethical committee of the Zhongshan hospital. All patients signed written informed consent to donate their tissue samples for research. Fresh tumor and normal tissues were subjected to bisulfite sequencing PCR (BSP) and quantitative PCR (qPCR) experiments.

Genomic DNA was extracted from tissue samples using a QiaAmp DNA Mini Kit (Qiagen, Valencia, CA, USA) according to the manufacturer’s manual. The DNA sample quality and integrity were determined by the A260/280 ratio and agarose gel electrophoresis using Nanodrop2000 (Thermo Scientific, USA) and Horizontal Electrophoresis Systems (Bio-Rad, USA). The BSP primers were designed using online websites with customization, and all PCR products were approximately 400 bp. The CpGs we were interested in were designed at almost the middle of the PCR product. Additionally, 250 ng of genomic DNA was converted using an EZ DNA Methylation-Gold Kit™ (Zymo Research, USA) according to the manufacturer’s manual. Bisulfite PCR amplification was performed with KAPA Uracil+ PCR Ready Mix (KAPA Biosystems, USA) and BSP PCR primers, and the PCR conditions were optimized. The PCR product was directly sequenced on an ABI 3730× system (Thermo Scientific, USA) using the same primers as the BSP amplification. The results from direct sequencing were analyzed with Sequencing Scanner 2 (Thermo Scientific, USA) using C/(C + T) peak ratios to define a CpG site methylation rate for each CpG dinucleotide within the covered region.

### Gene expression experiments

Total RNA was isolated with an RNeasy Plus Mini kit (Qiagen, Valencia, CA, USA) with DNase I digestion, and cDNA was synthesized by using a PrimeScript RT Reagent Kit (TaKaRa, Japan) according to the manufacturer’s manual. PCR primers were designed using Primer3 online tools. Quantitative PCR was performed using SYBR GREEN (Bio-Rad, USA) on an Eco qPCR system (Illumina, USA). Target mRNA expression was compared between the samples by normalization to beta-actin (ACTB) mRNA expression.

## Results

### Methylation landscape of HCC

**Fig. 1:**
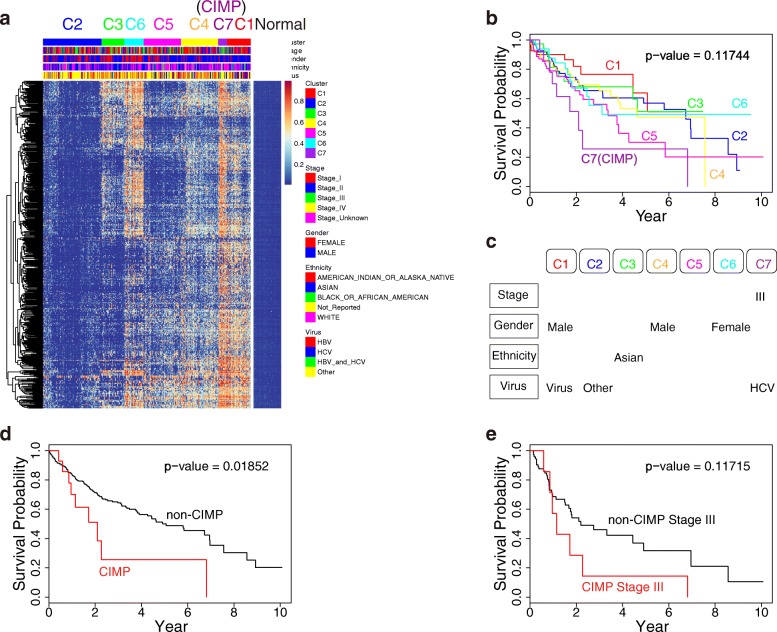
The DNA methylation landscape of hepatocellular carcinoma. **a** Seven methylation clusters were obtained from k-means consensus clustering. Rows are 591 CpGs that had high variation (SD > 0.2) in tumor tissues and low (β value < 0.05) methylation level in normal tissues. Cluster 7 (*purple*) showed a hypermethylation pattern in nearly all CpGs and was regarded as the CpG island methylator phenotype. **b** Kaplan-Meier survival curves of each cluster. The CIMP group had a poorer survival than other clusters. **c** Characteristics of the clusters. Significance was obtained from Fisher’s exact test (*p* value < 0.05). **d** Overall survival of CIMP and non-CIMP patients. **e** Overall survival of CIMP stage III and non-CIMP stage III patients

DNA methylation profiles of 375 HCC tumor samples and 50 adjacent normal tissue samples were obtained from TCGA. We selected the 591 most variable CpGs and performed unsupervised consensus clustering. HCC samples were classified into seven clusters (Fig. 1a). The methylation level of cluster 2 was the lowest. Cluster 7 (4.3%) showed widespread hypermethylation of promoter-associated CpGs and was considered to have the CpG island methylator phenotype. To determine whether the methylation subtypes are related to prognosis, the overall survival of each cluster was estimated using the Kaplan-Meier method. The *p* value obtained from the log-rank test is approximately 0.12, indicating there were differences in prognosis among the different subtypes (Fig. 1b). Furthermore, we compared the survival probability of CIMP patients (cluster 7) with those of other patients (clusters 1–6). The CIMP subgroup showed poorer prognosis (*P* = 0.0185; Fig. 1d).

We next examined whether the subtypes were significantly associated with clinical characteristics. The significant characteristics of cluster 1 were that there were more male (*P* = 0.0054) and virus-infected (HBV and HCV, *P* = 0.0012) patients. The genetic background of cluster 3 included mainly Asians (*P* = 0.0034). Cluster 2 had more patients without virus infection (*P* = 0.0055) and showed low methylation. Cluster 4 had more male patients (*P* = 1.21e-06) and cluster 6 had more female patients (*P* = 0.014). No significant characteristics were found for cluster 5. The CIMP group had more stage III (*P* = 0.0141) and HCV-infected (*P* = 0.0330) patients than the other clusters. To understand whether the poor prognosis of CIMP was due to more stage III patients, we compared the survival probability of stage III patients in the CIMP group and stage III patients in the non-CIMP group. We found that stage III patients in the CIMP group had a much poorer prognosis than stage III patients in the non-CIMP group (Fig. 1e). Hence, the poor prognosis of CIMP is possibly associated with global hypermethylation.

### Differential analysis of methylation and expression

**Fig. 2:**
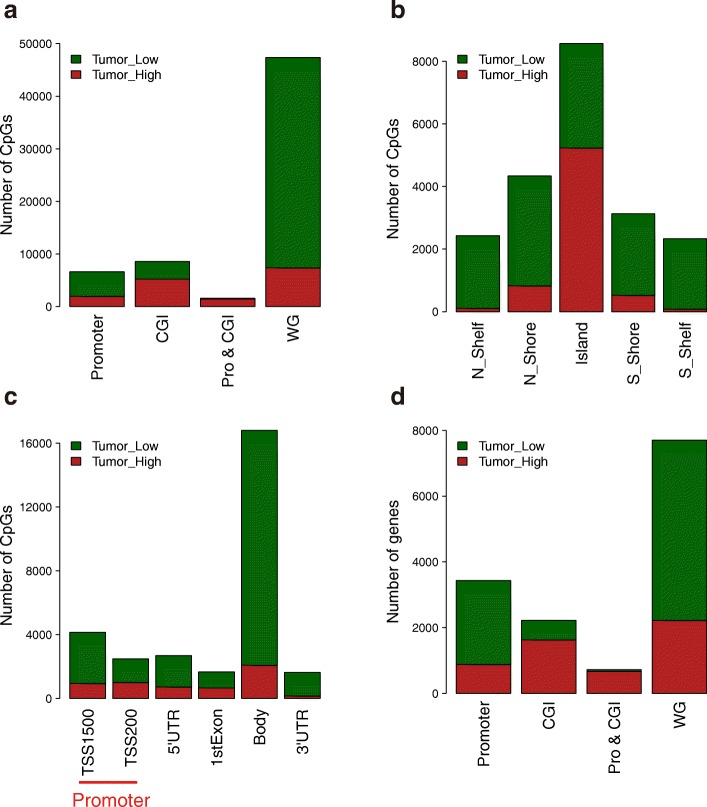
Distribution of differentially methylated CpGs and genes. **a** Distribution of differentially methylated CpGs in various genomic locations. *Promoter*, 1500 bp upstream of the transcription start site (TSS); *CGI*, CpG island; *Pro & CGI*, promoter and CpG island; *WG*, whole genome. **b** Distribution of differentially methylated CpGs according to CpG island. **c** Distribution of differentially methylated CpGs according to the distance to the TSS. **d**. Distribution of differentially methylated genes in various genomic locations

Methylation data of 50 paired samples from TCGA were used for differential methylation analysis (|β value difference| > 0.2 and FDR < 0.05). There were 7372 hypermethylated and 39,995 hypomethylated CpGs in HCC, which correspond to 2222 hypermethylated and 5478 hypomethylated genes. Then we analyzed the distribution of differentially methylated (DM) CpGs and genes in different genomic regions (Fig. 2a, d). Hypomethylation occurred globally in the whole genome, involving 84% of CpGs and 71% of genes. However, 61% of the CpGs (73% of genes) were hypermethylated in CpG-rich regions (CpG islands), and 91% of the CpGs (93% of genes) were hypermethylated in the CpG islands of the promoter regions. When we considered the distance of the probes to CpG islands, the percentage of hypermethylation was highest in the CpG island. This percentage decreased when the probes were far away from the CpG islands (Fig. 2b). The gene body was dominated by hypomethylation, while hypermethylation occurred preferentially in the regions around the transcription start sites (Fig. 2c). Such hypomethylation of the whole genome and hypermethylation of the promoter CpG islands are general characteristics of solid tumors. Expression data of 41 paired samples from TCGA were used for differential expression analysis (|log_2_ (fold change)| > 1 and FDR < 0.05). We found 662 highly expressed (“DE-high”) and 1553 lowly expressed (“DE-low”) genes in HCC.

### Roles of methylation in regulating gene expression

**Fig. 3:**
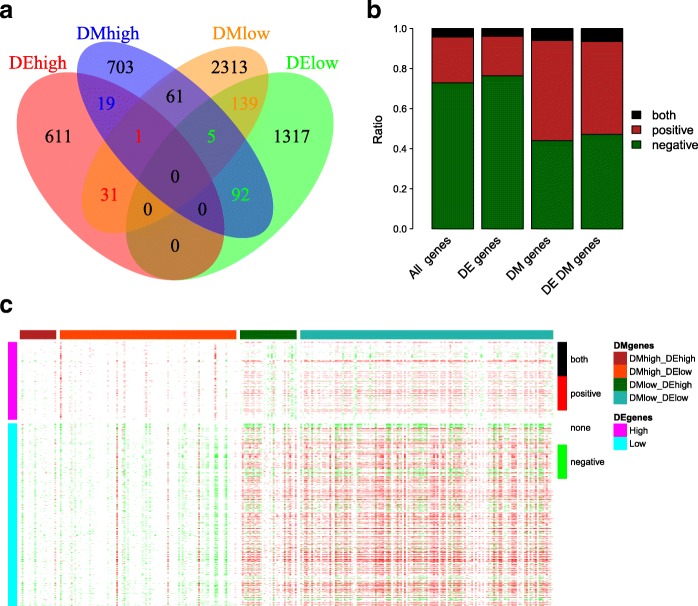
Relationship between DNA methylation and gene expression. **a** Comparison of differentially methylated genes and differentially expressed genes. Genes were considered differentially methylated if at least one promoter CpG site was significantly differentially methylated. **b** Correlation between gene expression and its promoter methylation. Correlations were calculated using all 16,206 genes, 2215 differentially expressed (DE) genes, 3364 differentially methylated (DM) genes, or 287 both DE and DM genes. The vertical axis shows the percentage of negatively correlated genes (*green*), positively correlated genes (*red*), and genes with both negative and positive correlation (*black*). **c** Correlation between promoter methylation and other gene expression. This analysis focused on the promoter methylation of 287 DM and DE genes (columns) and the gene expression of 2215 DE genes (rows). Positive and negative correlations are shown in *red* and *green*, respectively

First, we analyzed the intersection between differentially expressed genes and differentially methylated genes (Fig. 3a). Methylation alterations of the genes were assigned based on the status of the promoter methylation. Genes were called “DM-high” if at least one promoter CpG had a higher methylation level in HCC than in normal tissues. Similarly, “DM-low” genes had at least one hypomethylated promoter CpG. The promoter methylation defined 881 DM-high genes and 2550 DM-low genes. In total, 293 genes were differentially methylated and differentially expressed: 97 genes were hypermethylated with low expression in HCC, 32 genes were hypomethylated with high expression, 20 genes were hypermethylated with high expression, and 144 genes were hypomethylated with low expression (Fig. 3a). Since promoter hypermethylation plays important roles in the inactivation of cancer-related genes [28], we are particularly interested in the 97 highly methylated and lowly expressed genes, and in the subsequent analysis we used these genes to screen candidate diagnostic biomarkers.

To study the effect of DNA methylation on the expression of the same gene (*cis*-regulation), Pearson correlation coefficients were calculated between promoter methylation and gene expression. Among 16,206 genes with methylation and expression profiles, promoter methylation of 2798 (877) genes was significantly negatively (positively) correlated with gene expression (Fig. 3b). *Cis*-regulation was dominated by the negative correlation between promoter methylation and gene expression (Fig. 3b), which was consistent with previous reports [29, 30].

Furthermore, we investigated whether DNA methylation was related to the expression of other genes (*trans*-regulation). We focused on 512 CpGs in 287 differentially methylated and differentially expressed genes, analyzing their correlation with 2215 differentially expressed genes (Fig. 3c). The methylation of DM-high genes was predominantly negative correlated with gene expression while the methylation of DM-low genes was more likely to be positively correlated with gene expression.

### Identification HCC-specific methylation markers

**Fig. 4:**
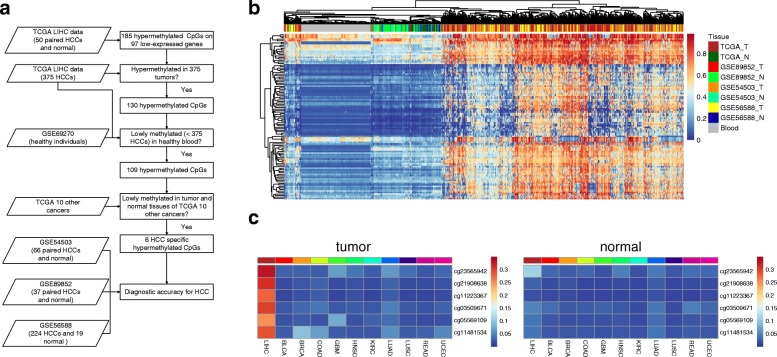
Identification of HCC-specific hypermethylated sites. **a** Protocol for finding candidate diagnostic biomarkers for HCC. **b** Unsupervised hierarchical clustering of HCC and normal controls using HCC hypermethylated sites. The heatmap shows the methylation levels of 109 CpGs in five datasets (TCGA, GSE54503, GSE89852, GSE56588, and GSE69270). Normal controls are clustered together, separated from HCC. **c** The average methylation level of six HCC-specific CpGs in HCC and ten other cancers

To find sensitive and specific methylation biomarkers for HCC, we designed a workflow to strictly screen biomarkers by comparing HCC with normal livers and other cancers (Fig. 4a). We started from 185 hypermethylated CpGs that were located in 97 lowly expressed genes. First, 130 CpGs remained after requiring hypermethylation in 375 HCC tissues. Second, the methylation data from blood of healthy people was used for filtering, and 109 CpGs were selected which were lowly methylated in healthy people and highly methylated in HCC. Figure 4b illustrates the methylation levels of these 109 CpGs in TCGA and three independent datasets (Additional file 1). Tumor samples could be well discriminated from normal tissue and blood samples, indicating the robustness of our results. Third, 109 CpGs were further filtered, requiring hypermethylation only in HCC but not in ten other cancers in TCGA, and six HCC-specific CpGs were obtained (Fig. 4c).

The six HCC-specific CpGs are mapped to four genes: NEBL (cg23565942), FAM55C (cg21908638, cg11223367, and cg03509671), GALNT3 (cg05569109), and DSE (cg11481534). Since the methylation status of CpGs is usually similar in neighboring regions [18], we investigated other CpG sites in the promoter of these four genes (Additional file 2: Figure S1). Most of the CpGs were also hypermethylated in HCC compared to normal tissues, consistent with the six specific CpGs. Next, we compared the methylation status of patients in different stages. The results showed that six HCC-specific CpGs are significantly hypermethylated even in stage I patients (Additional file 2: Figure S2). Therefore, these six CpGs are good candidates for the early detection of HCC.

### Evaluation of diagnostic accuracy in independent datasets

**Fig. 5:**
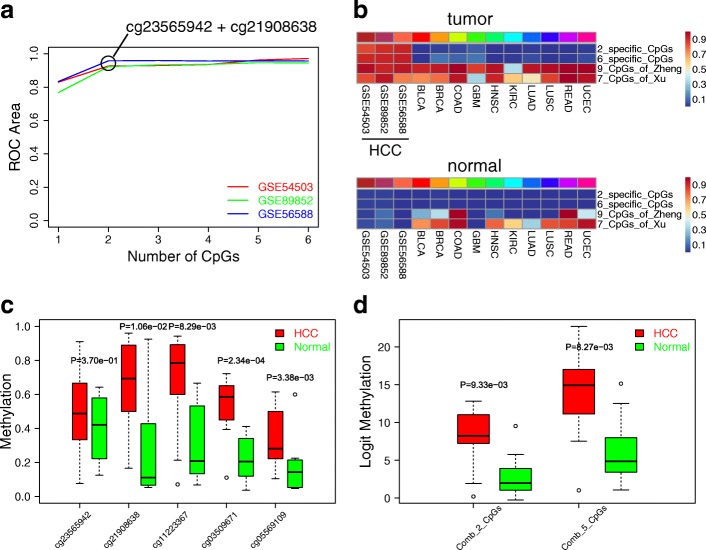
Performance of HCC-specific hypermethylated sites as diagnostic biomarkers. **a** Prediction accuracy using different combinations of HCC-specific CpGs. Logistic regression models were built using 50 paired TCGA samples and were tested using three independent datasets. Accuracy was measured by the area under the ROC curve. **b** Comparison of our markers with previously published methylation markers. Rows show different sources of methylation markers. The horizontal axis shows the different methylation datasets. The first three are HCC datasets, and the remainder are ten other cancer types. Colors indicate the percentage of different samples being predicted as HCC. **c** Validation of the methylation markers using ten paired HCC–normal tissues. Methylation values were measured by bisulfite sequencing PCR (BSP). **d** Combination score of methylation markers in ten paired HCC–normal tissues. Scores were calculated by the logistic regression model

Methylation data of the 50 paired HCC and normal tissues from TCGA were used as a training set. Three independent methylation datasets of HCC (GSE54503, GSE89852, and GSE56588) were used as test sets, including 327 HCC samples and 122 normal samples. Information gain-based feature selection was performed on the six CpGs to rank them. A logistic regression model was used to predict HCCs from one CpG to the combination of six CpGs. The ROC area associated with using one CpG to the combination of six CpGs to predict HCC in three independent datasets is shown in Fig. 5a. The performance using a combination of six HCC-specific CpGs was very good, with ROC areas of 0.972, 0.945, and 0.957 in GSE54503, GSE89852, and GSE56588, respectively. When using a combination of two specific CpGs (cg23565942 and cg21908638), the ROC area was higher than 0.92 in all three test sets. Hence, using a combination of two specific CpGs as markers could be more cost-effective.

Then, we compared our results with previously published methylation markers. Logistic regression models were built based on different feature sets: six or two CpGs from our study, nine CpGs from Zheng et al. [16], and seven CpGs from Xu et al. [17] (Additional file 3). The sensitivity and specificity of distinguishing HCC from normal livers were high and similar among the different feature sets (Table 1), while the number of CpGs we used was the least. Next, we compared the ability of different methylation markers to distinguish HCC from other cancers. Tumor and normal tissues from other cancers were seldom (0–12%, median 0.15%) predicted as HCC when using two or six HCC-specific CpGs in our study. However, 32.5 to 100% (median 92.85%) of tumor and 0 to 100% (median 48.95%) of normal tissues were predicted as HCC when using the CpGs of Zheng et al. and Xu et al. as feature sets (Fig. 5b). Therefore, our study found more cost-effective and specific biomarkers for HCC diagnosis.

**Table 1:** Comparison of the performance of different methylation markers for classifying HCC and normal tissues

Markers	Two HCC-specific CpGs	Six HCC-specific CpGs	Nine CpGs of Zheng et al.^a^	Seven CpGs of Xu et al.^b^
Sensitivity				
GSE54503	0.848	0.909	0.970	0.833
GSE89852	0.892	0.919	0.919	0.946
GSE56588	0.920	0.924	0.942	0.741
Specificity				
GSE54503	0.970	0.970	0.970	0.955
GSE89852	0.973	0.973	0.892	0.919
GSE56588	1.000	1.000	1.000	1.000

^a^Zheng et al. [16] reported ten CpGs as HCC diagnostic markers. Nine of them had methylation values in TCGA HCC dataset

^b^Xu et al. [17] reported ten CpGs as HCC diagnostic markers. Seven of them had methylation values in TCGA HCC dataset

To verify whether the six HCC-specific CpGs could be stably detected by cheaper technologies, BSP was used to determine the methylation status of ten fresh frozen HCC and normal tissues. The BSP primers of cg11481534 cannot amplify enough PCR products; thus, the methylation status of another five CpGs was analyzed (Additional file 4). Four specific CpGs (cg21908638, cg11223367, cg03509671, and cg05569109) were significantly hypermethylated (*P* < 0.05), as determined using the paired *t*-test. Another CpG (cg23565942) also showed some difference between the tumor and normal samples, although the *p* value (*P* = 0.37) was not significant (Fig. 5c). Then we combined the methylation of two specific CpGs and five specific CpGs according to the formula obtained from logistic regression and compared the difference in the combined score between the tumor and normal tissues. The combined score for the two and five CpGs was significantly higher in tumor tissues than in normal tissues, with *p* values of 0.009 and 0.008, respectively (Fig. 5d). Additionally, we validated the expression of the four genes mapped by six HCC-specific CpGs in the paired fresh frozen tissues by qPCR (Additional file 2: Figure S3). The expression of three genes (FAM55C, GALNT3, and DSE) was significantly (*p* < 0.05) lower in tumor tissues than in normal tissues. The expression of NEBL was also lower in the tumor, but the difference was not significant (*p* = 0.17). The phenomenon of hypermethylation and low expression of these genes in the fresh frozen tissues is concordant with that in TCGA HCC datasets. Thus, the specific CpGs identified in our study are promising diagnostic biomarkers specific for HCC.

## Discussion

In this study, we systematically analyzed the DNA methylation and gene expression data of hepatocellular carcinoma. We identified a subgroup of patients with CIMP and observed the poor prognosis of these patients. We found that methylation was negatively correlated with gene expression in *cis*-regulation. The patterns of *trans*-regulation are more complex; generally, the methylation of hypermethylated genes was negatively correlated with gene expression, while the methylation of hypomethylated genes was positively correlated with gene expression. Furthermore, we identified six CpGs as HCC-specific diagnostic biomarkers by comparing HCC, normal controls, and non-HCC cancers. These sites achieved ~ 91% sensitivity and ~ 97% specificity when predicting HCC. Our diagnostic biomarkers are more sensitive and more specific than most of the previously reported protein markers or methylation markers. These results provide new insights into the roles of DNA methylation in gene regulation and diagnosis.

CIMP is a phenomenon of simultaneous methylation of a group of genes in a subset of tumors [23] and has been studied in multiple cancer types, such as colorectal cancer (22.4%) [31], papillary renal-cell carcinoma (5.6%) [24], and glioblastoma (8.8%) [23]. It has been associated with prognosis, but the impact of CIMP on prognosis is not consistent among different cancers. We found that 4.3% of HCC patients had CIMP. Compared to other cancer types, the fraction of CIMP is smaller in HCC. However, the CIMP group needs special attention due to their poor prognosis. Somatic mutations of IDH1 and IDH2 have been reported to be associated with glioma CIMP [23]. Due to the low mutation frequencies of these genes in HCC, we did not observe a significant association between them and the CIMP group.

DNA methylation is an important epigenetic regulator of gene expression. We observed that *cis*-regulation is predominantly negatively correlated, which is concordant with the views of gene expression silenced by promoter DNA methylation [28, 32]. Promoter methylation of a hypermethylated gene was mainly negatively correlated with the expression of other genes, but promoter methylation of a hypomethylated gene was prone to being positively correlated with the expression of other genes. The reason for the inconsistent relationship of hypermethylated and hypomethylated genes in *trans*-regulation is unclear.

The most important finding of this study is the identification of several methylated CpGs as candidate diagnostic biomarkers of HCC. An ideal diagnostic biomarker should have high sensitivity, enabling the detection of HCC at an early stage; should be specific to HCC and not detected in other tumor types or premalignant liver diseases; should be measurable by non-invasive and cost-effective technology; and should be validated across different populations. Here, we discovered six HCC-specific hypermethylated sites whose sensitivity and specificity are better than the widely used serum biomarker AFP and another candidate serum biomarker, DKK1. Moreover, their methylation levels can be measured by relatively cheap PCR-based technology. However, we have not validated their diagnostic ability using non-invasive biospecimens. To resolve this problem, we will first develop a sensitive technology to detect methylation in cell-free ctDNA. Then, we will compare the consistency of methylation between tissues and blood and validate the prediction ability of the candidate biomarkers by measuring DNA methylation in the blood. Another problem is whether the methylation-based biomarkers could distinguish HCC from other liver diseases. In the future, we plan to investigate methylation profiles during the progression of liver cancers, including non-alcoholic fatty liver, hepatocirrhosis, and early HCC. Additionally, we are also interested in the downstream biological functions of methylation biomarkers, which may help us to understand the roles of methylation in carcinogenesis.

## Conclusions

DNA methylation plays important roles in gene regulation and carcinogenesis in HCC. We discovered several methylation-based biomarkers by analyzing the genome-wide methylation data of 375 HCC samples, 50 normal liver samples, 3780 samples of cancers of other sites, and 474 normal samples of other organs. The candidate biomarkers were validated in three independent datasets with more than 300 HCC samples and 100 normal liver samples. Then, BSP-based experimental validation was performed in ten HCC patients. The candidate biomarkers achieved high diagnostic ability and have the potential to be translated into clinical application. Future translational research will accelerate the clinical validation of candidate biomarkers and promote the early detection of HCC. A similar analysis method could be used for other tumor types to find more associations between methylation and cancer diagnosis.

## Additional files

Additional file 1: Methylation information for 109 CpGs in different HCC datasets. (XLSX 26 kb)

Additional file 2: **Figure S1.** Promoter methylation of four genes. **Figure S2** Stage-related methylation of six HCC-specific CpGs. **Figure S3** Gene expression validation of the four genes by qPCR. (PDF 2167 kb)

Additional file 3: Prediction performance of specific CpGs in distinguishing HCC from normal tissue and other tumors. (XLSX 12 kb)

Additional file 4: Primers of CpGs or genes in BSP and gene expression experiments. BSP and qPCR data of fresh frozen tissues from ten HCC patients. (XLSX 14 kb)

## Abbreviations

AFP: Alpha-fetoprotein
BSP: Bisulfite sequencing PCR
CIMP: CpG island methylator phenotype
ctDNA: Circulating tumor DNA
DCP: Des-γ-carboxy prothrombin
DE: Differentially expressed
DKK1: Dickkopf-1
FDR: False discovery rate
GEO: Gene Expression Omnibus
HBV: Hepatitis B virus
HCC: Hepatocellular carcinoma
HCV: Hepatitis C virus
PCR DM: Differentially methylated
TCGA: The Cancer Genome Atlas
TSS: Transcription start site
