# Supplementary material for: Integrative analysis of DNA methylation and gene expression reveals hepatocellular carcinoma-specific diagnostic biomarkers
Source: Genome Med. 2018 May 30;10:42. doi: 10.1186/s13073-018-0548-z (PMC5977535; doi:10.1186/s13073-018-0548-z)
Supplement: Supplementary file 2 — Figure S1. Promoter methylation of four genes. Figure S2 Stage-related methylation of six HCC-specific CpGs. Figure S3 Gene expression validation of the four genes by qPCR. (PDF 2167 kb) [file 13073_2018_548_MOESM2_ESM.pdf]

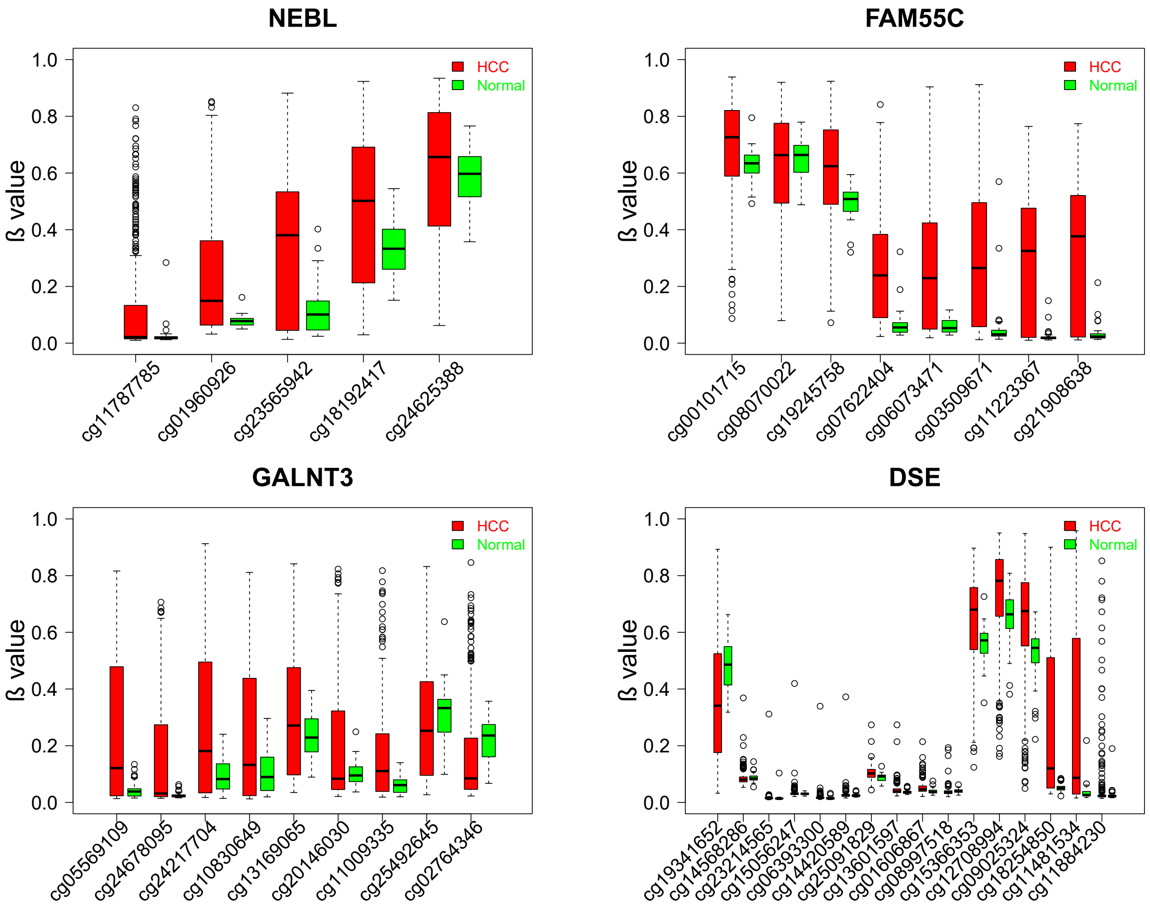

Fig. S1 Promoter methylation of four genes.

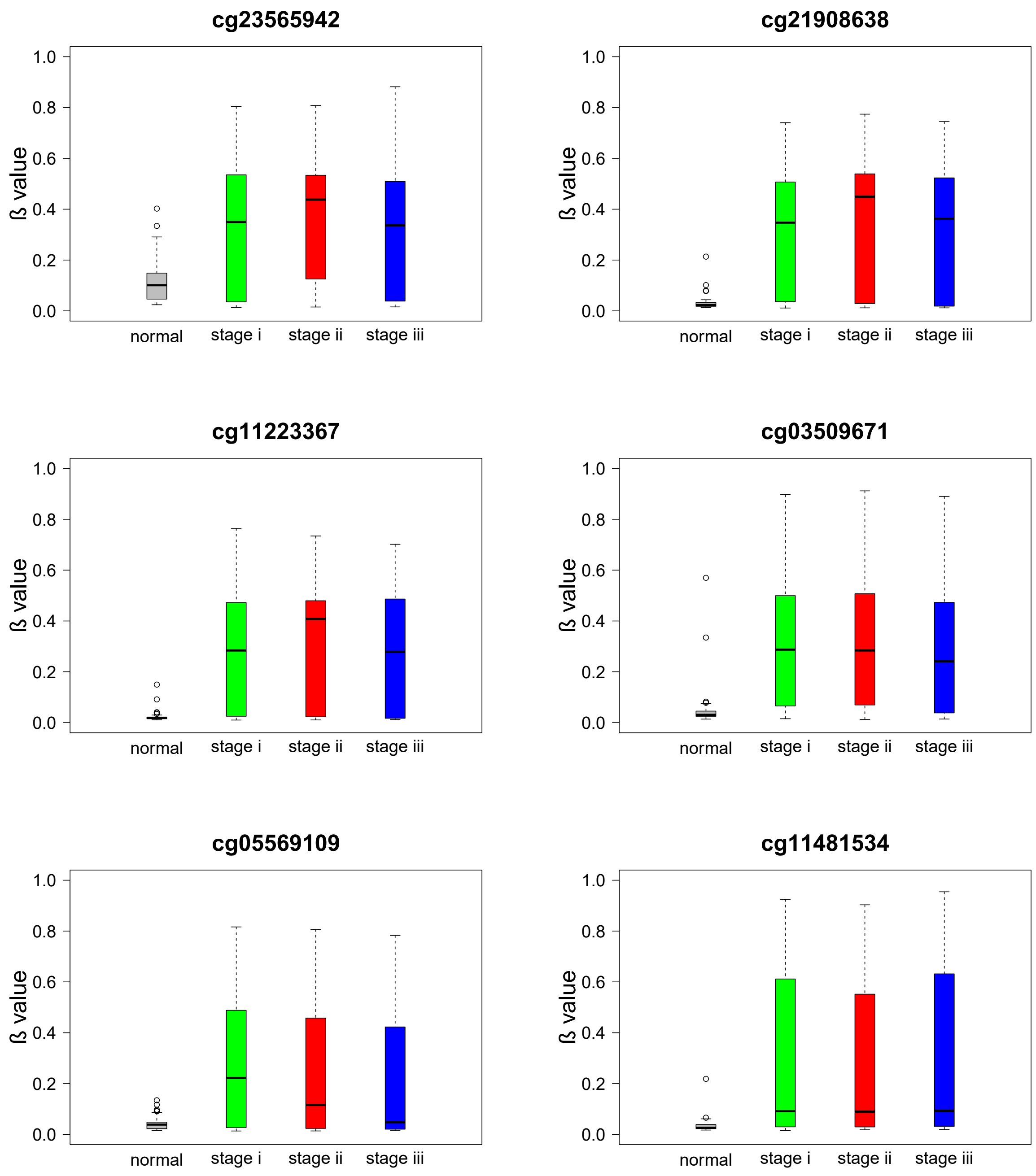

Fig. S2 Stage related methylation of six HCC-specific CpGs.

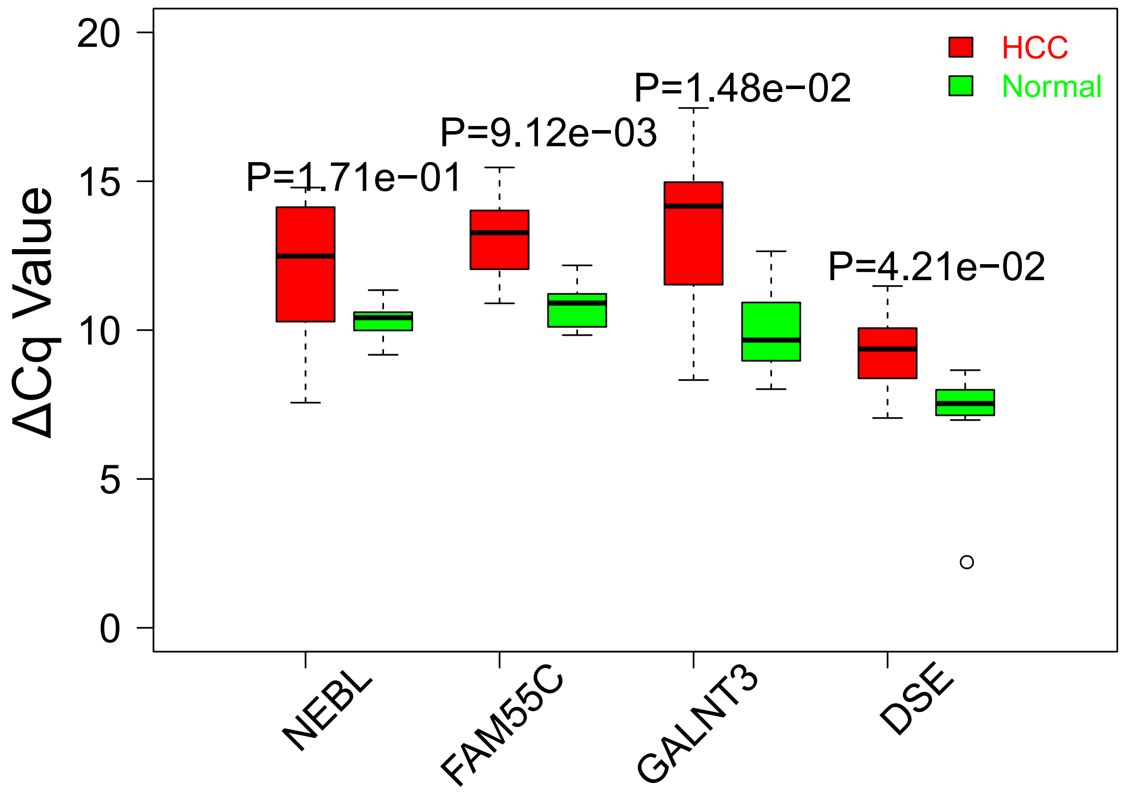

Fig. S3 Gene expression validation of the four genes by qPCR. Δ Cq value of each gene was obtained by subtracting the raw-Cq value of this gene from that of the reference gene ACTB. The ΔCq value of a gene is inversely proportional to the gene expression. Cq: quantification cycle.
